# Vegetables, Potatoes and Their Products as Sources of Energy and Nutrients to the Average Diet in Poland

**DOI:** 10.3390/ijerph18063217

**Published:** 2021-03-20

**Authors:** Hanna Górska-Warsewicz, Krystyna Rejman, Joanna Kaczorowska, Wacław Laskowski

**Affiliations:** Department of Food Market and Consumer Research, Institute of Human Nutrition Sciences, Warsaw University of Life Sciences, 07-787 Warsaw, Poland; krystyna_rejman@sggw.pl (K.R.); joanna_kaczorowska@sggw.pl (J.K.); waclaw_laskowski@sggw.pl (W.L.)

**Keywords:** vegetables, potatoes, energy supply, nutrient supply, food sources

## Abstract

The aim of our study was to analyse vegetables, potatoes and their products as sources of energy and nutrients in the average diet in Poland. Representative data of the 2016 Household Budget Survey from 36,886 households were used. This is the largest study sample in Poland, so we generalized the conclusions to the whole population using the statement ‘average diet’. We analysed three main product groups: vegetables, vegetable products, and potatoes and potatoes products, dividing them into 14 subgroups (e.g., tomatoes, cabbage, carrots, other vegetables, and mushrooms). The percentages of energy, protein, carbohydrates, total fat, nine vitamins (thiamine, riboflavin, niacin, vitamin B6, folate, vitamin C, vitamin A, vitamin D, and vitamin E), seven minerals (calcium, phosphorus, sodium, potassium, iron, magnesium and zinc), and fibre from the analysed food subgroups are presented. Additionally, the influence of household characteristics on the supply of energy and nutrients from each subgroup of vegetables, potatoes, and their products was evaluated using cluster analysis. In the analysis, R programme and Kohonen neural networks were applied. Our study showed that vegetables, potatoes, and their products provide 7.3% of daily dietary energy supply. Vegetables contribute more than 20% of the supply of six nutrients: vitamin C (51.8%), potassium (32.5%), folate (31.0%), vitamin A (30.6%), vitamin B6 (27.8%), and magnesium (20.2%), as well as fibre (31.8%). Cluster analysis distinguished three clusters that differed in nutritional supply from vegetables, potatoes, and their products. Educational level, income measured by quintile groups, village size, socio-economic characteristics, urbanization degree, and land use were the most important factors determining differences between clusters.

## 1. Introduction

Vegetables and potatoes are important staple foods consumed daily worldwide. Vegetables are considered as an essential component of a healthy diet, so nutrition experts recommend eating at least a few servings of them every day [1,2,3,4,5,6]. Potatoes are characterised by a large scale of production, consumption, affordability with easy availability in the open market and therefore are a vital food-security crop [7,8,9]. On the other hand, the literature describes many factors that determine the level and structure of the consumption of vegetables, potatoes, and their products [10,11,12,13,14]. The nutritional value of vegetables and potatoes differs considerably and should therefore be considered separately.

### 1.1. Vegetables: Nutrition Value and Health Benefits

For nutrition purposes, the term ‘vegetable’ refers to a plant cultivated for its edible part(s) or refers to the edible parts of a plant [15,16]. These can be roots (for example beet, carrot, radish), underground buds with stems covered with layers of leaves (garlic, leek, onion), leaves (spinach, lettuce, endive), flowers (broccoli, cauliflower, globe artichoke), fruits (tomato, cucumber, snap beans), or seeds (green peas) [17]. Their nutritional composition differs depending on particular species, varieties and other factors, such as growing and storage conditions, product maturity and processing (i.e., fresh, frozen, dehydrated, etc.) [18]. Overall, vegetables are an important source of vitamins (especially C, A, B1, B6, B9, E), minerals (particularly potassium, calcium, magnesium) and dietary fibre. They also contain various beneficial bioactive compounds including polyphenols (mainly found in leaves and flowers), carotenoids (in different vegetables with yellow, orange, red, or purple pigmentation) sulphur compounds (present in some species in the underground buds with roots and stems covered with leafy layers), and phytosterols [16,19,20,21]. Concerning macronutrients, some vegetables may contain significant amounts of protein (e.g., green peas, fresh broad beans, Brussels sprouts) and carbohydrates (beets, sweet kernel corn, non-dried; baby corn, parsnips). Most vegetables are low in fat and high in water content, making them naturally low in calories [17].

The epidemiological studies have confirmed that certain plant compounds as phytochemicals and vitamins are powerful antioxidants, protecting against free radicals damage and modify metabolic activation as well as detoxification of carcinogens [15,22]. People whose diets are rich in vegetables have a lower incidence of certain types of cancer (colon, pancreas, prostate, mouth, pharynx, larynx, oesophagus, stomach, lung, etc.) [23,24,25,26,27,28,29] and other serious chronic diseases (such as heart disease, stroke, hypertension, diabetes, etc.) [6,23,28,30,31,32,33,34,35]. It is easier for them to maintain a suitable body mass [4,6,21]. Other vegetable nutrients such as minerals (e.g., potassium, calcium, magnesium) contribute to blood pressure regulation and reduce the risk of stroke [21]. Certain plant components, i.e., dietary fibre, phytochemicals and vitamins lower cholesterol and prevent the oxidation of cholesterol and other lipids in the arteries and increase the formation of endothelial prostacyclin that inhibits platelet aggregation and reduces vascular tone [36,37]. Finally, a unique combination of minerals and phytochemicals linked to the dietary fibre matrix improves bowel transit and helps to control blood glucose and may also help reduce calorie intake [6,19,21].

While there are important health benefits to eating vegetables, most of the world’s population is not consuming the recommended amount due to the interaction of different factors including food production, supply and affordability, access, food environments, and individual consumption behaviours [10,11]. 

To help prevent nutrition-related chronic diseases, experts from the World Health Organization (WHO) and the Food and Agriculture Organization of the United Nations (FAO) recommend a minimum individual intake of 400 g of fruit and vegetables per day, excluding potatoes and other starchy tubers [2,38]. In 2015, 81 countries representing around 55% of the world’s population (mostly from high-income countries in Europe, North America, and East Asia-Pacific) had an average availability of fruit and vegetables above the WHO minimum target. When considering more stringent age-adjusted recommendations, only 40 countries, representing about 36% of the global population, had the sufficient availability to supply the recommended intake level [11]. This dietary guideline is promoted worldwide as “5 a day” or “5 a day the colour-way” which refers to eating five servings of 80 g of various fruits and vegetables every day. In some countries, even higher amounts are recommended, such as 500 g per day in Sweden, 600 g in Denmark, 650 to 750 g in Norway, and 640 to 800 g in the US. Relevant governmental campaigns have been launched to promote greater fruit and vegetable consumption, such as “5 a Day” in England, France, Germany, and many other European countries, “Fruit and Veggies—More Matters” in the US, which recommended eating different numbers of servings of fruits and vegetables depending on individual calorie needs, “Go for 2 & 5” in Australia, which promoted two servings of fruit (150 g per serving) and five servings of vegetables (75 g per serving) per day [22,23]. Finally, a range of global action plans have been developed to prompt initiatives to increase fruit and vegetable intake, including the WHO Global Strategy on Diet, Physical Activity and Health, the WHO Global Action Plan for the Prevention and Control of Non-Communicable Diseases 2013–2020, and the United Nations Decade of Action on Nutrition 2016–2025 [10,11]. The EAT-Lancet Commission promotes a model of a planetary diet, healthy for people and the environment, in which almost half of the daily food intake, in units of weight, is fruits and vegetables, with starchy vegetable (tubers, potatoes, cassava) being a separate group and augmenting the total intake of fruits and vegetables [39].

### 1.2. Potatoes: Nutrition Value and Health Benefits

Potatoes are the third most important crop in the world (in terms of food supply) for human consumption, after wheat and rice [15,40,41]. Botanically, they are classified as perennials in the Solanaceae family, grown for their starch-rich tubers that form at the ends of underground shoots [17]. Carbohydrates are the dominant nutrient of potatoes. They constitute about 75% of the total dry weight of the tuber, mainly in the form of starch, the amount of which varies depending on the variety. The late-maturing cultivars tend to produce much greater starch yield compared to the early-maturing cultivars. A slight proportion of starch is resistant to enzymatic degradation in the stomach and small intestine, and therefore has similar physiological effects and health benefits for the microbiota as dietary fibre [21,42]. Potatoes contain a small amount of protein, but its biological value is high due to the presence of essential amino acids such as lysine and metabolites that can increase its utilization [21]. Potatoes contribute considerable amounts of vitamins C and B6, and trace amounts (thiamin, riboflavin, folate, and niacin). Dietary fibre is found in potatoes in an amount of 0.5 to 2%, half of which is found in the flesh. Potatoes are a staple food for many populations, including Poland, which is why they are listed among the food groups that are the main sources of dietary fibre [43]. They are also a good source of minerals, including potassium, magnesium, phosphorus, and iron. Phytochemicals in potatoes, mainly carotenoids and phenolic acids, are predominantly found in the coloured varieties i.e., yellow, red, and purple. Processing and meal preparation methods do impact the bioavailability and loss of certain nutrients in the potatoes, particularly vitamin C and potassium. The nutrient loss appears to be greatest when potatoes are boiled in water and/or treated at high temperatures for a long period (e.g., baking) [42]. Potatoes may also contain toxic glycoalkaloids (two major examples are α-chaconine and α-solanine), which serve to protect the tuber from pathogens, insects, parasites, and predators but can lead to gastrointestinal disorders in humans such as nausea, vomiting, abdominal cramping, or diarrhoea [44]. Their levels vary greatly among cultivars and may increase after harvest, due to environmental factors such as light, mechanical damage and storage. Nevertheless, removing the sprouts and peeling of the skin before processing removes almost all of the glycoalkaloids [7,42]. On the other hand, peeling leads also to a partial loss of dietary fibre and bioactive compounds [45].

Due to nutritional properties, potatoes may play a role in preventing micronutrient deficiencies and reducing the risk of certain chronic diseases such as high blood pressure, heart disease, cancer, neurodegenerative diseases, and diabetes. This is mainly due to the presence of vitamins, minerals, and phytochemicals with free radical scavenging properties and the fact that they are a common part of the diet of people around the world [41]. 

On the other hand, eating potatoes may increase the risk of weight gain (which is associated mainly with the consumption of processed potato products, particularly chips and French fries), and type 2 diabetes due to the carbohydrate content resulting in a high glycaemic index [42]. Potato preparations made at high temperature under certain conditions (presence of high amounts of reducing sugars and asparagine), mainly French fries and other deep-fried preparations contain the carcinogenic acrylamide [7]. Since the detection of acrylamide in food in 2002, many national and international regulatory agencies have taken initiatives to understand its formation in fried and baked products and to reduce its presence in foods intended for human consumption [46].

Potatoes play an important role in preventing malnutrition, especially in poorer parts of the world, and in view of the growing population and demand for food [9,41]. In food-deficit and low-income areas of the world, potato consumption is increasing rapidly due to rising prices for other staple foods (wheat, rice, and maize) and the high nutritional value of potatoes [7,8]. As potatoes are not demanding in cultivation and are characterized by a high production scale, affordable price and easy availability on the market, they are a vital crop in achieving food security. Trends in global potato consumption show that, while consumption is steadily declining in Europe, there is accelerated growth in Africa and Asia [47,48]. Their accessibility contributes to the achievement of several sustainable development goals, e.g., SDG 1: no poverty, SDG 2: zero hunger, and SDG 12: responsible consumption and production [7,8,9,49].

Considering the above arguments, the aim of our study was to analyse vegetables, potatoes, and their products as sources of energy and nutrients in an average diet in Poland. This survey was conducted by Statistics Poland on the representative sample of the Polish population. This study is the next stage of our research relating to sources of energy and nutrients in food consumption patterns in Poland. Previously, separate publications have analysed the role of five other food categories in the nutritional value of the diet [50,51,52,53,54] and the sources of two nutrients from the food consumed [55,56]. 

## 2. Methods

### 2.1. Study Description

The aim of our study was to analyse the contribution of vegetables, potatoes and their products in the provision of energy and nutrients in an average diet in Poland. We examined carbohydrates, protein, total fat, seven minerals, nine vitamins as well as fibre, and considered 14 food subgroups from three main product groups: vegetables, vegetable products, and potatoes and potato products. 

### 2.2. Data Collection

Our study is based on the database of the representative Household Budget Survey (HBS) conducted systematically by Statistics Poland (SP). In 2016, the survey sample was 36,886 households (resulting in 99,230 individuals) [57]. In a two-stage household selection procedure, area survey points were drawn first, followed by households for each such point. In 2016, 1586 area points were drawn, including 911 urban and 665 rural points. Households were drawn separately for each point proportionally. In each household, records were kept monthly in the “Household Budget Diary”. Data were collected in the “Household Statistics Sheet” [57,58]. The research method has been systematically improved as a method for assessing food consumption [59]. This is the largest study sample in Poland, so we generalized the conclusions to the whole population using the statement ‘average diet’. We have detailed the sampling procedure in our previous publications [50,51,52,53,54,55,56].

The quantitative consumption of 91 food subgroups in each household was converted to per capita intake per month. The next step was to recalculate the intake data into energy, macronutrients, vitamins, and minerals for the 91 subgroups. For these calculations, we used “Nutritive Value Tables for Foods and Meals” (4th ed.) [60]. We then calculated the average supply of energy and nutrients in weight units and as a percentage of the contribution to the total diet. 

### 2.3. Food Grouping 

We analysed three main groups and 14 subgroups related to vegetables, potatoes, and their products (Table 1). This classification is based on the assumptions made to divide all food products into main groups and subgroups [50,51,52,53,54,55,56]. The classifications used in the literature take into account the structure of product consumption in a given food category. For example, in the UK National Nutrition Survey, the category ‘vegetables and potatoes’ is divided into four groups [61]. 

### 2.4. Statistical Analysis

For a more detailed analysis, the influence of household characteristics on the supply of energy and nutrients to the average diet was examined using cluster analysis [62,63] and the Kohonen neural network [64,65] in Statistica 13.3 software (Copyright 1984-2917, TIBCO Software Inc., Palo Alto, CA, USA). The sample population was divided into three clusters based on *p* < 0.05000. This is the most optimal number of clusters due to a large number of classification variables, i.e., 280 variables resulting from the study of 20 nutrients and 14 items comprising the categories of vegetables, potatoes, and their products studied. Each cluster has been characterized with 14 demographic, social, and economic factors, including education level, income (quintile group), socio-economic household affiliation, degree of urbanization, size of the village or city, region, family life phase, land usage, financial and nutrition self-assessment, age, gender, survey month, and household size. The impact of each factor was tested using the Cramer’s correlation index. For each cluster, we presented detailed characteristics for those factors for which the value of the Cramer correlation index was significant.

### 2.5. Data Presentation

We present the results in Section 3 in the following order:

- Vegetables, potatoes and their products as sources of energy and macronutrients—Section 3.1.
- Vegetables, potatoes and their products as sources of minerals—Section 3.2.
- Vegetables, potatoes and their products as sources of vitamins—Section 3.3.
- Cluster analysis: the influence of household characteristics on the supply of energy, minerals and vitamins from the food groups analysed—Section 3.4.

## 3. Results

Sources of energy, macronutrients and dietary fibre (Table 2), minerals (Table 3), and vitamins (Table 4) from vegetables, potatoes, and their products are presented below.

### 3.1. Vegetables, Potatoes and Their Products: Supply of Energy, Macronutrients and Fibre

Vegetables, potatoes, and their products provided 7.3% of energy to the average diet in Poland (Table 2), including potatoes with 3.5% of the energy supply and other vegetables and mushrooms with 1.3%. 

The analysed product groups supply 31.8% of the fibre, of which vegetables accounted for 18.2%, potatoes and potatoes products accounted for 9.1%, and vegetable products accounted for 4.5%. Among vegetables, other vegetables and mushroom, carrots, and tomatoes provided the most fibre. 

The supply of carbohydrates from vegetables, potatoes and their products amounted to 12.6%, of which the largest share was held by potatoes and their products (7.3%), followed by vegetables (4.1%). Protein was supplied by vegetables, potatoes, and their products in the amount of 7.9%. Vegetables accounted for the largest share of this amount—4.2%—and potatoes and their products accounted for 2.7%.

### 3.2. Vegetables, Potatoes and Their Products: Supply of Minerals 

The supply of seven minerals from vegetables, potatoes, and their products is presented in Table 3. These products are responsible for the highest supply of potassium, providing 32.5% of this mineral in the diet. Exactly half of this amount is supplied by potatoes and their products (16.2%), of which potatoes alone account for 15.1%. Vegetables supply 13.2% of potassium, with the highest share of other vegetables and mushrooms (5.2%) as well as tomatoes (3.1%).

Vegetables, potatoes, and their products supply magnesium and iron at a similar percentage level, accounting for 20.2 and 19.0% of the average contribution, respectively. The share of vegetables in magnesium contribution amounted to 9.6%, and of potatoes and their products 8.5%. On the other hand, the share of these product groups in iron supply was 11.1 and 5.5%, respectively. Among the subgroups analysed, potatoes contributed the most magnesium (7.9%), followed by other vegetables and mushrooms (5.2%). In the case of iron supply, these two subgroups also stood out, but in reverse order (other vegetables and mushrooms—6.1%, potatoes—5.0%).

The contribution of vegetables, potatoes, and their products in the supply of zinc and phosphorus remained in the range 12 to 13%. For zinc, vegetables came first (7.9%), followed by potatoes and potato products (3.6%). For phosphorus, the share of vegetables and potatoes and their products was at a similar level of 5 to 6%. For these minerals, potatoes and other vegetables and mushrooms were also dominant sources.

### 3.3. Vegetable, Potatoes, and Their Products: Supply of Vitamins 

The contribution of vegetables, potatoes, and their products to the supply of vitamins is presented in Table 4. The largest share of the examined product category in the supply of vitamins was obtained for vitamin C (51.8%), of which the share of vegetables was 30.6%, potatoes and potato products was 14.1%, and vegetable products was 7.1%. In the group of vegetables, the most vitamin C was provided by other vegetables and mushrooms (10.0%), tomatoes (7.4%), and cabbage (7.0%). The share of cauliflower was also remarkable (2.4%). In the group of potatoes and their products, the main contribution to vitamin C supply came from potatoes (13.4%). 

The second highest supply to the average Polish diet by vegetables, potatoes, and their preserves was folate (31.0%). Vegetables provided 21.4% of the total folate supply, including the share of other vegetables and mushrooms at the level of 8.4% and tomatoes at 4%. On the other hand, potatoes and their products accounted for 6.1% of folate supply and vegetable products for 3.5%.

The contribution of vegetables, potatoes, and their products to the vitamin A supply in the average diet was very similar to that of folate, at 30.6%. Vegetables had the highest share of 25.7%, with carrots dominating (16.3%). Vegetable products supplied 4.6% of vitamin A, including a considerable share of other vegetable and mushrooms products of 3.0%, and frozen vegetables and mushrooms contributing 1.7%. 

In the supply of vitamin B6, the share of vegetables, potatoes, and their products was 27.8%, with the highest share of potatoes and their products (15.3%). Vegetables accounted for 10.0% of this vitamin supply, of which other vegetables and mushrooms had a major share with 4.1%, and tomatoes with 2.4%. 

For the other vitamins we studied, the share of vegetables, potatoes, and their products in the supply to the average diet ranged from 17.1% (thiamin) to 11.3% (riboflavin). In the supply of thiamin, the share of vegetables was 9.0%, while that of potatoes and their products was 6.6%. In the case of niacin, potatoes and their products were of greatest importance (8.6%), followed by vegetables (6.2%). In the supply of riboflavin and vitamin E, vegetables were most important, representing 6.8 and 8.1% of the daily supply, respectively. In the group of vegetables, tomatoes ranked first in terms of vitamin E supply, and in terms of riboflavin, other vegetables and mushrooms ranked first.

### 3.4. Cluster Analysis

To summarize our results, the impact of the household characteristics on the supply of energy and nutrients supply from vegetables, potatoes, and their products was analysed using cluster analysis. Education level, income expressed in quintile groups, the village size, socio-economic type of the household, urbanization degree, and land use were the most determining factors for the issues studied (Table 5). The sample population was divided into three clusters based on *p* < 0.05. The characteristics of these clusters, taking into account the criteria selected during the analysis, are presented in Table 6. 

The largest cluster (cluster 1), comprising almost 56% of the number of households, was characterised by the lowest share of energy supply and all examined nutrients (except fat) from vegetables, potatoes, and their products to the average diet in Poland (Table 7). For example, vegetables, potatoes, and their products provided 51.8% of vitamin C, 31.0% of folate, and 30.6% of vitamin A, whereas in cluster 1, these values were, respectively, 45.1, 25.7, and 25.9%. Cluster 2, which encompassed 27.1% of the surveyed households, was characterised by the highest proportion of energy (10.9%) and seven nutrients: carbohydrates (19.5%), phosphorus (17.2%), potassium (45.9%), magnesium (28.2%), niacin (23.5%), vitamin B6 (41.0%), and vitamin C (65.2%) provided by the analysed food category. In contrast, in cluster 3, comprising the smallest number of households (17.2% of the total sample), the contribution of vegetables, potatoes, and their products to the supply of nutrients was highest for twelve of them, namely: protein (12.0%), fat (2.8%), calcium (14.5%), sodium (5.3%), iron (27.5%), zinc (20.1%), thiamine (24.7%), riboflavin (17.0%), folate (43.8%), vitamin A (43.1%), vitamin D (39.5%), and vitamin E (18.7%).

**Figure 1:**
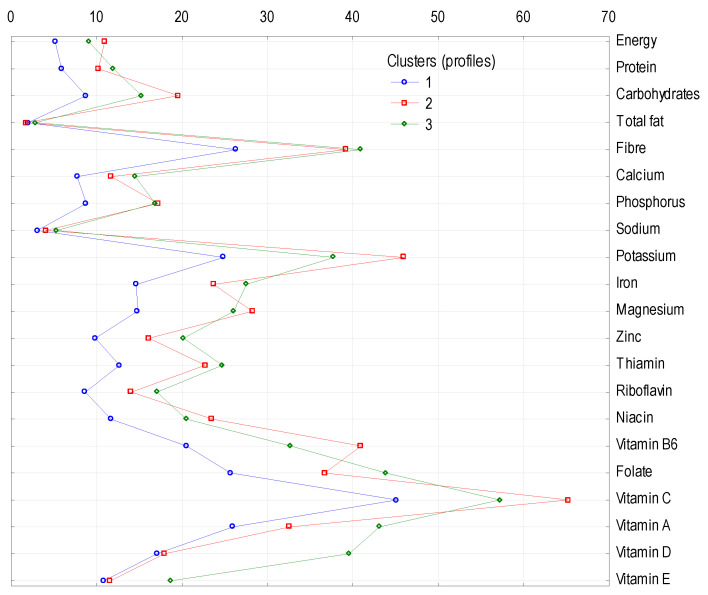
Cluster analysis: percentage contribution of selected nutrients from vegetables, potatoes, and their products.

**Figure 2:**
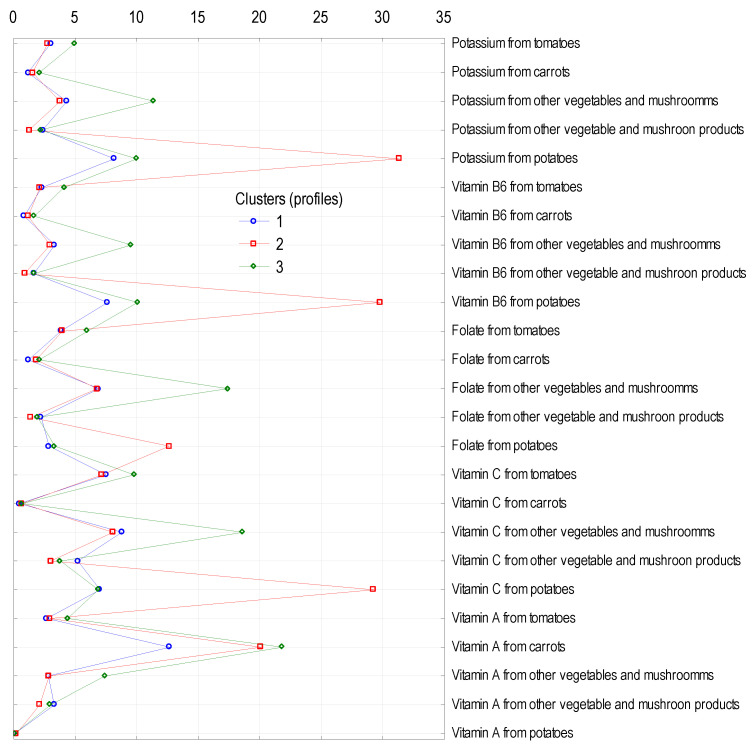
Cluster analysis: percentage contribution of selected nutrients from selected vegetables, potatoes, and their products.

Figure 1 compares the role of the analysed food category in providing energy, macronutrients, minerals, and vitamins to the populations forming the clusters. Figure 2 shows the importance of potatoes in providing potassium, vitamin B6, folate, and vitamin C for people in cluster 2 and of carrots as a source of vitamin A (in the form of carotenoids) for people in each cluster.

## 4. Discussion

The aim of our study was to analyse the vegetables, potatoes, and their products as sources of energy and nutrients in the average diet in Poland based on the representative 2016 HBS. In the analysis, we considered three main groups and 14 subgroups of this category of food. We compared our results with the other studies published in the scientific literature on the Australian [66], Spanish [67,68], American [69,70], Korean [71,72], UK [61], and Dutch [73] populations. Such a comparison was made for energy and those nutrients whose supply in the Polish diet is 20% and more.

### 4.1. Vegetables, Potatoes, and Their Products as Sources of Energy in the Average Diet in Poland

Our research has indicated that vegetables, potatoes, and their products supplied 7.3% of energy, of which potatoes provided 3.5% (together with preparations 4.0%), vegetables provided 2.5%, and vegetable products provided 0.9%. These data are comparable to the results obtained for the Australian population. Studies conducted among adults aged 19 years and more indicate that vegetable products and dishes provided 8.3% energy (women) or 9.0% energy (men). The energy supply from potatoes amounted to 5.8 to 5.6% [66]. The data concerning the average American diet indicate lower values for the energy provided by potatoes (2.9%) [69]. Similarly, in the case of Americans ≥ 51 years old, the value of energy supplied by vegetables (5.9%) was lower than in Poland [70]. In the Dutch diet, the energy supply from vegetables was 2%, whereas potatoes and other tubers supplied 5% [73]. The situation is different for the UK diet. Vegetables and potatoes supply 10.5% of energy (adults aged 19 to 64) and 10.6% (adults ≥ 65 years); for salads and other raw vegetables, the value amounted to 0.7 to 0.9%, while for chips, fried and roast potatoes and potato products the value was 4.2 to 3.2%; and for other potatoes, potato salads and dishes it was 1.7 to 2.9% [61]. The comparisons presented are difficult to assess because the assumptions and research methodology are different in each country. Secondly, the proportion of vegetables as energy sources or other nutrients is determined by dietary patterns, which differ between populations. In the UK diet, both groups of potatoes provide 5.9 to 6.1% of dietary energy, and these amounts are comparable to the Australian and Dutch dietary data. The populations of these countries follow a typical dietary pattern for highly developed countries. In Poland, on the other hand, potatoes are a staple food, consumed mainly in the cooked form in a variety of dishes, so the contribution of processed potatoes to energy supply is negligible. The smallest share of potatoes in the energy of the diet of an average consumer in the United States seems to contradict the stereotypical perception of an American diet, with lots of French fries. But the annual consumption of potatoes in this country is the lowest among the compared countries (according to Food and Agriculture Organization, FAO data, it was 55.3 kg in 2018; in Poland, it was the highest—99.5 kg) [74], and since the early 1970s, the consumption structure has been dominated by processed potatoes [75]. Fresh potatoes account only for 29% of the potatoes grown in the US and are used primarily for baked, boiled, or mashed potatoes (2015 to 2019 average). Processed potato products have become the major mover in the potato market. The market of manufactured potatoes is led by frozen products, which accounts for 44% of the potato harvest, of which 85% is intended for the production of French fries, one of the most popular convenience foods consumed all over the world. Chipping potatoes use 16% of US potato production [76] and other frozen potato products (such as Tater Tots, spiral fries, homefries, wedges, and frozen whole potatoes) use a smaller percentage [77]. The growth of the U.S. potato processing industry is driven by an increase in disposable income and growth in fast food outlets, coupled with an increase in the penetration of online shopping and home delivery services. The North American fries market forecast by age group indicates that the segment of 20 to 35 years old consumers is the most prominent, accounting for the maximum market share and representing the highest consumption growth rate. This can be attributed to the habit of frequent snacking by people of this age. Moreover, the majority of this demographic group includes working millennials, who prefer consuming more convenience, packaged or ready-to-eat meals due to their busy lifestyle [78]. The average American currently eats more than 13 kg of fries every year [75]. However, the negative impact of fried food on human health acts as a major restraining factor for this market. Innovations in the market, including low carbohydrate French fries or air fried/baked fries, are expected to provide growth opportunities for this market [78].

### 4.2. Vegetables, Potatoes, and Their Products as Sources of Dietary Fibre in the Average Diet in Poland

From the total number of 20 food components (including 19 nutrients and dietary fibre), eight were provided by the discussed category of food in the amount exceeding 20% of their total supply in the average diet in Poland. These were: fibre (31.8%), vitamin C (51.8%), folate (31.0%), vitamin A (30.6%), vitamin B6 (27.8%), vitamin D (20.8%), potassium (32.5%), and magnesium (20.2%). We chose to include iron in the discussion because 19.0% of this nutrient came from the food category analysed, as well as providing almost ¼ of the iron intake for individuals in cluster 2 and more than ¼ in cluster 3 (23.7 and 27.5%, respectively). 

Vegetables, potatoes, and their products are an essential source of dietary fibre in the diet in Poland, accounting for 31.8% of fibre contribution. Other vegetables and mushrooms had the highest share (6.9%), followed by potatoes (8.3%), other vegetable and mushroom products (2.8%), carrots (2.6%), and tomatoes (2.0%). In terms of the total fibre supply, a similar value was recorded for the average UK diet (30% for persons aged 19 to 64 and 32% for persons over 65). However, the structure of supply was different: salad and other raw vegetables supplied 4.0% of fibre; vegetables (not raw) including vegetable dishes supplied 16.0%; chips, fried and roast potatoes, and potato products supplied 7.0 to 5.0%; and other potatoes, potato salads and dishes supplied 4.0 to 6.0% [61]. A lower fibre supply from vegetables, potatoes, and their products was observed in the average Australian diet (26.4% in women’s and 26.9% in men’s diets). Only potatoes supplied a similar amount as in Poland (9.5 to 9.1%). Other sources of fibre in the Australian diet include cabbage, cauliflower, and similar brassica vegetables (2.8 and 3.2%), carrot and similar root vegetables (2.7 and 2.9%), leaf and stalk vegetables (1.5 and 2.0%), peas and beans (3.8 and 3.5%), tomato and tomato products (1.7 and 2.0%), other fruiting vegetables (1.4 and 1.9%), and other vegetables and vegetable combinations (2.8 and 2.8%) [66]. A slightly lesser importance of this product category was observed in the Dutch diet: vegetables provided 14% of energy, while potatoes and other tubers provided 10% [73]. Among the compared diets, the lowest supply of fibre from vegetables and potatoes occurred in the average American diet (18.9%), of which potatoes supplied 6.4% of fibre, other vegetables supplied 5.5%, tomatoes and tomato/vegetable juice supplied 4.6%, and corn, peas, and lima beans provided 2.4% [69]. On the other hand, among Americans aged 51 years and older, the share of vegetables in the supply of fibre was slightly lower at 18.3% [70].

In addition to vegetables, fruit and cereal products provide dietary fibre. Its content in foods is generally low, so it must be collected from the many different plant products consumed each day. Eating a variety of food, both relatively high and low in fibre can help to achieve a relatively high fibre diet [79]. The fibre content per 100 g of vegetables ranges from 0.5 to 5.8 g, while in fruit it is lower, averaging about 2 g per 100 g of fruit. In the average Polish diet, cereal products are the source of almost half of the fibre consumed [52] and vegetables are the second source, as shown in our analysis. The third source is fruit, with the share of vegetables remaining constant at about 1/3 of the total fibre supply [80]. The daily intake of fibre among adult women in Poland is 19.4 to 20.0 g/person, among men it is 25 to 34 g/person [81], and among vegetarians of both sexes it reaches an average of 60 g/person [82]. According to international recommendations, the consumption of 25 g of fibre per day is necessary for the proper functioning of the organism [1].

### 4.3. Vegetables, Potatoes, and Their Products as Sources of Vitamins in the Average Diet in Poland 

Vitamin C. The sources of vitamin C in the human diet are fewer than in the case of fibre; they are exclusively vegetables and fruits. In the average diet in Poland, vegetables, potatoes, and their products are an important source of vitamin C, providing more than 50% of this vitamin. A similar result was found in the diet in Spain, where the percentage of vegetables in the vitamin C supply was 50.6% [67]. As our study indicated, potatoes (13.4%), other vegetables and mushrooms (10.0%), tomatoes (7.4%), and cabbage (7.0%) have a large share in the supply structure of this vitamin. The proportion of vegetable products and dishes (adults 19 years and over) in the Australian diet was at a lower level of 40.3% for women and 40.8% for men. The structure of the supply of this vitamin was also different: potatoes (13.7 and 11.4%); cabbage, cauliflower, and similar brassica vegetables (10.8 and 12.2%); tomato and tomato products (4.8 and 5.1%); other fruiting vegetables (5.1 and 5.9%) [66]. The comparison with average diets in other countries shows even lower rates of vitamin C; e.g., in the Dutch diet, the proportion of vegetables in the supply of vitamin C was 16%, while for potatoes and other tubers it was 13% [73], and in the average diet of Americans aged 51 years and over, it was 25.6% [70]. The differences are mainly due to the availability of fresh fruit and the level of fruit consumption in the compared countries. In the Netherlands and the United States, fruit consumption is much higher, at food balance levels of 100 and 90 kg/person, respectively [74], so more vitamin C may come from this food group. Although vitamin C is one of the most labile, potatoes are an important source of vitamin C, especially in countries with a high intake of them, such as Poland. The monthly average consumption of potatoes in Polish households in 2016 was 3.48 kg/person, followed by processed foods (0.90 kg) and tomatoes (0.83 kg) [57].

Folate. Our study indicated that vegetables, potatoes, and their products provide 31% of folate, with other vegetables and mushrooms delivering 8.4%, potatoes 5.7%, tomatoes 4.0%, and cabbage 2.8%. Comparing our data with other diets, a higher value was found for the Korean diet; the proportion of vegetables in the folate quantity was 39.9% [71]. In turn, lower values were obtained for the average Australian, Danish, American, and British diets. In the average Australian diet, the share of vegetable products and dishes was 26.7% for women and 29.6% for men. The folate supply structure was as follows: potatoes (8.1 and 9.1%); cabbage, cauliflower, and similar brassica vegetables (4.6 and 5.6%); carrot and similar root vegetables (2.3 and 2.6%); leaf and stalk vegetables (3.2 and 4.3%); peas and beans (2.8 and 2.9%); tomato and tomato products (1.5 and 1.8%); other fruiting vegetables (1.7 and 2.3%); and other vegetables and vegetable combinations (2.3 and 2.6%) [66]. The UK dietary folate content of vegetables and potatoes was 26.0% in the 19-to-64-year-old population and 28.0% in the over 65 year old population, with the following items in particular: salad and other raw vegetables (7.0%); vegetables including vegetable dishes (12 and 14%); chips, fried and roast potatoes, and potato products (4 and 2%); other potatoes, potato salads and dishes (3 and 5%) [61]. Diets from the other countries we compared were characterized by a lower share of vegetables and potatoes in the folate supply. For example, in the average Dutch diet, the contribution of vegetables to folate intake was 5%, while for potatoes and other tubers it was 10% [73]. In the average diet of Americans aged 51 years and older, the share of vegetables was 9.5% [70]. Folate, similarly to vitamin C, can be classified as an exogenous vitamin (only small amounts come from internal synthesis) and the most labile one, especially sensitive to high temperature, oxygen, sunlight, acidic environment. Studies indicate that the efficiency of folate absorption from food does not exceed 50%, which is why women of childbearing age are recommended to supplement this vitamin [83]. Among food groups, vegetables have the highest folate content, especially those from the brassica family—including broccoli, Brussels sprouts, and kale—as well as parsley and parsley root, other leafy vegetables, and pulses [60]. In household budget surveys in Poland, the latter are included in the subgroup of other vegetables; thus, among others, its contribution to the dietary folate supply was the highest. In the compared diets of different countries, the importance of potatoes in the provision of folates was confirmed. Additionally, in some of them, the importance of cabbage/brassica vegetables and tomatoes was also confirmed.

Vitamin A. Our research indicates that vegetables, potatoes, and their products provide 30.6% of vitamin A. Vegetables and fruits are rich sources of it, especially carrots, pumpkin, red peppers, chicory, parsley, spinach, kale, and broccoli (as well as fruits with yellow or orange flesh). More than half of vitamin A came from carrots (16.3%), followed by other vegetable and mushrooms (3.5%) and their products (3.0%), frozen vegetables and mushrooms (1.7%), and tomatoes (1.1%). Comparing these data with the results for other diets, it is worth noting the higher proportion of vegetables and their processing in the average Australian diet, at 35.3% for women and 38.6% for men. In this value, carrots and similar root vegetables had a significant share of 21.9 and 23.7%. These data show the greater importance of carrots in providing vitamin A compared to the average diet in Poland. The next place was taken by other fruiting vegetables with 5.5 and 6.5%. The share of other vegetables was at the level of potatoes (1.8 and 1.6%), and tomato and tomato products (1.7 and 1.9%) [66]. Similarly, higher rates for vegetables were obtained in the average Korean diet, including such items as sweet potato (23.3%), carrot (12.0%), spinach (8.3%), lettuce (4.1%), pumpkin (3.6%), kimchi (2.3%), perilla (2.0%), watermelon (1.2%), and welsh onion (1.0%) [72]. Comparable values to those obtained in our study were noted in the average UK diet. The share of vegetables and potatoes in the average diet was 28.3% for people aged 19 to 64 years and 32.7% for people over 65, including salad and other raw vegetables (7.8 and 7.0%), vegetables and vegetable dishes (19.8 and 25.5%), and chips, fried and roast potatoes, and potato products (0.5 and 0.2%) [61]. In the other diets studied by us, we observed a lower supply of vitamin A from vegetables amounting to 15% in the average Dutch diet [73] and 23% in the average diet of Americans aged ≥ 51 years old [70] 

Vitamin B6. Vegetables, potatoes, and their products are a good source of vitamin B6, providing 27.8% of the total daily supply. More than half of this amount is provided by potatoes, and other notable sources are processed vegetables (including sauerkraut), tomatoes, and carrots. Similar data were obtained during the study of the Korean diet, as the percentage of vegetables in the vitamin B6 supply was 28.8% [71]. Data from other countries indicate lower values, 16.3% for vegetables in the Spanish diet [68], 5% for vegetables and 19% for potatoes and other tubers in the Dutch diet [73], as well as 11.7% for vegetables in the American diet of adults aged ≥ 51 years [70]. Vitamin B6 is fairly common in both animal and plant foods, and in the food category under review, potatoes, tomatoes, red peppers, and sauerkraut are good sources. Eating raw vegetables, in particular, is important in terms of supplying the body with vitamin B6 since it is lost through heat treatment, most significantly through cooking. Eating sauerkraut salads, very popular in Poland, additionally strengthens the body with a portion of probiotics.

Vitamin D. Concerning the dietary intake of vitamin D, it should be emphasized that, for humans, food is a supplementary source of this vitamin since about 80% of its intake comes from the process of endogenous synthesis. Small amounts of vitamin D are contained in food of animal origin. Hardened vegetable fats are enriched with this vitamin (in Poland, in the case of margarine, this is an obligatory practice). This may explain the symbolic share of processed potatoes in providing this vitamin to the Polish diet. Mushrooms, fresh or marinated, are the source of almost the entire amount of this vitamin from food (which are included in the discussed product category), and since recently the Polish industry also offers frozen mushrooms. In Poland, it is customary to pick mushrooms in forests, which are eaten fresh and stored for later use in the form of home-made pickles or dried. Mushrooms are an ingredient of many dishes, regional or prepared for special occasions. Ergocalciferol is found in mushrooms—1.49 µg/100 g in fresh ones, and 1.34 µg/100 g in marinated ones [60].

### 4.4. Vegetables, Potatoes, and Their Products as Sources of Minerals in the Average Diet in Poland 

Potassium. In the average diet in Poland, the share of vegetables, potatoes, and their products in the potassium supply is 32.5%, of which the most important are potatoes (15.1%), other vegetables and mushrooms (5.2%), and tomatoes (3.1%). In the compared diets, we observed a lower share of vegetables and potatoes in the potassium supply. For example, in the Australian diet (adults 19 years and over), the share of vegetable products and dishes in the potassium supply was 24.4% for women and 25.5% for men. In the detailed structure, the share of potatoes deserves attention (13.8 and 12.4%), while tomato and tomato products (2.0% and 2.4%), and cabbage, cauliflower and similar brassica vegetables (1.9 and 2.3%) were of lesser importance [66]. The share of vegetables and potatoes in the supply of potassium at a similar level was recorded in the average UK diet (24 to 26%), with particular emphasis on such products as chips, fried and roast potatoes and potato products (9% for people aged 19 to 64 years and 6% for people over 65 years), other potatoes, potato salads and dishes (5 and 8%), salad and other raw vegetables (3 and 4%), and vegetables including vegetable dishes (7%) [61]. The lower supply of potassium from vegetables and potatoes was observed in the average Dutch diet (vegetables—9%; potatoes and other tubers—11%) [73]. On the other hand, in the American diet, the proportion of potatoes amounted to 6.7%, tomatoes, tomato/vegetable juice to 5.9%, and other vegetables to 3.4% [69]. In turn, in the average diet of Americans at the age of 51 years and above, vegetables provided 15.7% of potassium [70]. Potassium is found in almost all food groups, with vegetables being a very good source and potatoes slightly worse. However, a high consumption of potatoes determines a high intake of potassium, as our study showed for the diet in Poland. Potassium does not receive much public attention in the prevention of diet-related diseases, yet the decreasing potato consumption in Poland and the relatively stable vegetable consumption may result in worsening deficits of this nutrient. Potato consumption in Polish households has decreased by 46% over the last ten years (from 5.07 to 2.72 kg/person/month). Consumption has only partially shifted to the foodservice sector, as food balance sheets show a 20% drop in potato consumption over the same period. Today, the majority of people in the world consume a diet relatively high in salt and low in potassium. A high dietary sodium to potassium ratio is associated with hypertension, cardiovascular disease, and all-cause mortality [84].

Magnesium. Magnesium is, next to potassium, the most important intracellular cation, but unlike potassium, it is not so commonly found in foods. In the food category analysed, potatoes are rich in magnesium and only some vegetables, especially green vegetables, because magnesium is part of chlorophyll. Pulses are also a good source of magnesium, but their share is marginal in food consumption in Poland. In the average Polish diet, the supply of magnesium from vegetables, processed vegetables and potatoes was 20.2%, of which vegetables supplied 9.6% and potatoes 8.5%. A similar proportion of these food groups as a source of magnesium was reported in the Australian diet (adults aged 19 years and over). Vegetable products and dishes provided 12.8% of magnesium for women and 13.7% for men, while potatoes provided 6.4% and 5.8%, respectively [66]. In the average American diet (adults aged ≥ 51 years), vegetables provided 9.1% of magnesium [70]. A lower magnesium supply from the studied food category was noted in the average British diet (16 to 17%). In the detailed list, magnesium supply achieved the largest share for vegetables (not raw) including vegetable dishes (7.0%), followed by chips, fried and roast potatoes and potato products (4 to 5%), other potatoes, potato salads and dishes (3 to 5%) as well as salad and other fresh vegetables (2.0%) [61]. In the average Dutch diet, the supply of magnesium from vegetables and potatoes and other tubers was even lower, amounting 5 and 6% of the total intake of this nutrient [73]. This does not necessarily mean a magnesium deficit in the Dutch or British population, as cheese, cocoa, and chocolate or bananas are also good sources of magnesium. The appropriate composition of the daily diet can ensure the supply of this mineral, but it should be borne in mind that only half of the amount taken from the diet will be absorbed. Magnesium intake in Poland is deficient, as shown by many studies [85].

Iron. In the average diet in Poland, vegetables and potatoes provided 19.0% of iron, of which vegetables provided 11.1%, while potatoes provided 5.5%. By comparison, in the average UK diet, vegetables and potatoes provided slightly less iron (15%), including vegetables (not raw) and vegetable dishes (8 to 9%), chips, fried and roast potatoes and potato products (2 to 3%), other potatoes, potato salads and dishes (2 to 3%), and salads and other fresh vegetables (2%) [61]. Lower values were recorded for the average Australian diet (adults aged 19 years and over). Vegetable products and dishes provided 11.7% of iron for women and 12.9% for men. The supply of iron for the other items studied in this product category was as follows: potatoes (11.7 and 12.9%, respectively), cabbage, cauliflower and similar brassica vegetables (1.2 and 1.5%, respectively), as well as peas and beans (1.3 and 1.1%) [66]. The lowest values were observed for the Dutch and American diets. In the average Dutch diet, vegetables provided 8% of iron, while potatoes and other tubers provided 5% [73]. In contrast, in the average American diet (adults aged ≥ 51 years), vegetables provided 5% of the iron [70]. The iron in plant foods is nonheme iron, which is sensitive to both inhibitors and enhancers of iron absorption. The inhibitors include phytates, calcium, and the polyphenolics in tea, coffee, herb teas, and cocoa. Fibre only slightly inhibits iron absorption. Some food preparation techniques (soaking and sprouting beans, grains, and seeds, and the leavening of bread) can diminish phytate levels and thereby enhance iron absorption. The group of enhancers includes vitamin C and other organic acids as well as fermentation processes [86]. The incidence of iron-deficiency anaemia among vegetarians is similar to that of non-vegetarians [87]. More than half of the vitamin C in the Polish diet is provided by the food category analysed, so it substantially enhances iron absorption and reduces the inhibitory effect of phytates, thereby improving the iron status. Among vegetables, parsley has the highest iron content, but apart from beetroot and potatoes mentioned above, chard, parsley root, kale, kohlrabi, green peas, leek, spinach and other leafy vegetables are also good sources of iron. Pulses, in Poland, classified as other vegetables, have an iron content comparable to parsley and in plant-based diets provide significant iron intake.

### 4.5. Recommendations for Improving Dietary Habits

Analysis of the importance of vegetables, potatoes, and their products in the provision of nutrients and dietary components showed significant variation between clusters. In cluster 1, which includes every second person and every second household, we found the lowest contribution of these foods to the provision of all items. Based on the characteristics in Table 6, people in this cluster can be identified as primarily employees and pensioners, and residents of smaller towns. In terms of the level of education, they ranked between cluster 3 (with the best education, 10% with basic vocational and lower education) and cluster 2 (60% with basic vocational and lower education), and the income of the majority (60%) was average or lower. This is practically the profile of a “statistical Pole”. In this population group, vegetables, potatoes, and their products provided more than 20% of the daily intake of only six dietary components, while in the general population it was seven components in cluster 2–10 and in cluster 3–11 components. In cluster 1, the analysed food category provided too little iron, magnesium, vitamin B6, folate, vitamin C, and vitamin A compared to the other clusters. Data from the Household Budget Survey 2016 [59] show that vegetable consumption in the fifth quintile group of households is 60% higher than in the first quintile group, and potato consumption is only 20% lower. They also show that residents of large cities eat more vegetables than those from smaller cities and rural areas.

The cited data and facts indicate that there is an urgent need to intensify nutrition education in Poland, in order to convince the population to eat more vegetables, and in general to change their diet to a plant-based diet. These changes are part of the urgent need to change dietary patterns to healthy and sustainable ones, which is included in the development strategy for the European Union’s common agricultural policy for the coming years. This is a necessary action to achieve the goal set out in the European Green Deal, which is to achieve climate neutrality for the European region by 2050.

In Poland, new dietary guidelines for healthy eating were presented in October 2020, illustrated graphically in the form of a plate, instead of the previous pyramid of healthy eating [88]. The plate is filled with food groups, the size of which symbolises the recommended proportions of each group in the daily diet. The subtitle of the plate is “Eat a variety of foods every day”. In addition, around the plate there are three categories of recommendations—“Eat less”, “Eat more”, and “Swap”—in which the individual product groups are listed. The graphic also includes the need for daily physical activity and maintaining a healthy body weight.

Half of the plate is filled with fruit and vegetables. Although the proportions are not specified, visually about 2/3 of this half is vegetables. In the “Eat more” prompts, there is a recommendation for a higher intake of “different coloured vegetables and fruit—more vegetables than fruit”. The leaflet outlining the new dietary guidelines shows how the recommendation to eat more fruit and vegetables can be achieved in three steps: (1) eat a vegetable or fruit at every meal, (2) eat a minimum of 400 g of fruit and vegetables every day—more vegetables than fruit, (3) eat as many different-coloured fruit and vegetables as possible—each extra serving of fruit and vegetables is a further health benefit.

Achieving a significantly higher intake of fruit and vegetables compared to the current dietary pattern in Poland is a huge challenge, but it is consistent with the direction of the further development of food systems and the model of healthy sustainable food consumption or the so-called planetary diet of the EAT-Lancet Commission [39]. Diets based on a wide variety of nutrient-rich local plant foods, including vegetables, that contain moderate amounts of animal protein (preferably in the form of fish) and are low in saturated and trans-fatty acids, added sugars and sodium, are healthy, nutritious, sustainable and climate-friendly [89]. However, in the new Polish “plate for healthy eating” there is not a single reference to sustainability or concern for the environment (which is included in the dietary recommendations in many other countries), while one could expect a synergistic effect by combining health and environmental factors.

It is essential to develop, fund, and implement a national nutrition education strategy, at all levels, from government to pre-school, using all methods and tools to motivate people to change their dietary habits. Activities should both support more informed and desirable food choices and aim to change the food market environment. Creating an environment where such diets are also economically advantageous and convenient may be a part of a global solution to current nutritional challenges [89].

## 5. Conclusions

Knowing the nutritional composition of the products in the food category analysed, it is obvious that only potatoes and some vegetables provide protein and carbohydrates, and therefore energy in the diet. However, our research has shown the important role of the analysed food category, i.e., vegetables, potatoes, and their products in providing many valuable micronutrients. Potatoes, whose consumption in Poland, despite a high decline, is still high, turned out to be an important source of potassium (15%), vitamin B6 (14%), vitamin C (13%), fibre, magnesium and niacin (8% each), folate (6%), phosphorus and iron (5% each). The role of vegetables in the diet is also to provide non-nutritive components with functional effects, including antioxidants, which are important in the prevention of diet-related diseases. The postulated dietary changes to a more sustainable, plant-based diet will increase the importance of vegetables in providing nutrients and other health-promoting substances and will benefit in improving public health indicators. International and national nutrition science societies, based on scientific evidence, have taken the position that appropriately planned vegetarian diets, including total vegetarian or vegan diets, are healthful, nutritionally adequate, and may provide health benefits in the prevention and treatment of certain diseases. This regards individuals during all stages of the life cycle. However, the need to reduce meat consumption in favour of vegetables and other foods of plant origin, for health and environmental reasons, is encountering barriers, both from consumers (mainly for hedonic reasons) and from meat producers. It is therefore necessary to use all possibilities and at all levels to reach out to all consumer groups. Our study showed that there are differences between households in the amount of consumption of vegetables, potatoes, and their preparations (including pulses), which are determined especially (with statistical significance) by the education level, income, and socio-economic affiliation of the household, as well as several characteristics of the place of residence.

## Figures and Tables

**Table 1:** Food groups analysed in the study.

Main Groups	Subgroups
Vegetable	(1) Lettuce (2) Cabbage (3) Cauliflower (4) Tomatoes (5) Cucumber (6) Carrots (7) Beetroot (8) Onions (9) Other vegetables and mushrooms
Vegetable preserves	(10) Frozen vegetables and mushrooms (11) Sour cabbage (12) Other vegetable and mushroom preserves
Potatoes and potato products	(13) Potatoes (14) Potato products

**Table 2:** Vegetables, potatoes, and their products as sources of energy, macronutrients, and fibre in the diet.

Specification	Energy	Protein	Carbohydrates	Total Fat	Fibre
**Average daily supply**	**2,261,00 kcal**	**77.90 g**	**270.37 g**	**96.91 g**	**17.64 g**
**Supply of vegetable, potatoes and their products**	**16,573 kcal**	**6.18 g**	**34.01 g**	**1.98 g**	**5.86 g**
**Share of vegetables, potatoes and their products in average daily supply (%):**	**7.33**	**7.93**	**12.58**	**2.03**	**31.80**
**Vegetables:**	**2.45**	**4.17**	**4.14**	**0.64**	**18.19**
lettuce	0.04	0.09	0.06	0.01	0.42
cabbage	0.17	0.34	0.31	0.04	1.89
cauliflower	0.04	0.11	0.07	0.01	0.48
tomatoes	0.25	0.34	0.46	0.06	2.03
cucumbers	0.10	0.15	0.17	0.02	0.40
carrots	0.19	0.17	0.43	0.03	2.64
beetroots	0.09	0.13	0.18	0.01	0.73
onions	0.23	0.23	0.43	0.04	1.26
other vegetables and mushrooms	1.33	2.62	2.04	0.40	8.34
**Vegetable products:**	**0.92**	**1.11**	**1.13**	**0.82**	**4.51**
frozen vegetables and mushrooms	0.11	0.17	0.19	0.02	1.03
sour cabbage	0.04	0.08	0.08	0.01	0.72
other vegetable and mushroom products	0.77	0.85	0.86	0.79	2.77
**Potatoes and potato products:**	**3.96**	**2.65**	**7.31**	**0.58**	**9.10**
potatoes	3.45	2.32	6.65	0.11	8.25
potato products	0.52	0.33	0.66	0.47	0.85

The values in the columns do not add up because each time the conversion is done from the whole population according to the R environment rule. Differences are in hundredths of decimal places as a result of calculations from a database with many thousands of records. Data in bold are cumulative for the entire population or summed for individual groups, i.e., vegetables, vegetable products, and potatoes and potatoes products.

**Table 3:** Vegetables, potatoes, and their products as sources of minerals in the diet.

Specification	Calcium	Phosphorus	Sodium	Potassium	Iron	Magnesium	Zinc
**Average daily supply**	**644.10 mg**	**1160.19 mg**	**3863.84 mg**	**2617.85 mg**	**10.28 mg**	**267.33 mg**	**9.78 mg**
**Supply from vegetables, potatoes, and their products**	**62.09 mg**	**141.54 mg**	**147.60 mg**	**849.50 mg**	**1.95 mg**	**53.87 mg**	**1.27 mg**
**Share of vegetables, potatoes, and their products in average daily supply (%):**	**9.35**	**12.2**	**3.82**	**32.45**	**19.00**	**20.15**	**12.98**
**Vegetables:**	**6.89**	**5.72**	**2.25**	**13.18**	**11.05**	**9.6**	**7.88**
Lettuce	0.19	0.09	0.01	0.26	0.35	0.17	0.23
Cabbage	1.36	0.37	0.05	1.21	0.58	0.68	0.46
Cauliflower	0.12	0.11	0.02	0.31	0.22	0.17	0.09
Tomatoes	0.42	0.52	0.06	3.05	1.39	0.85	0.77
Cucumbers	0.37	0.29	1.09	0.59	0.36	0.99	0.60
Carrots	0.79	0.36	0.29	1.36	0.62	0.77	0.45
Beetroot	0.46	0.09	0.07	0.66	0.84	0.39	0.33
Onions	0.54	0.15	0.55	0.58	0.60	0.44	0.31
Other vegetables and mushrooms	2.65	3.74	0.11	5.15	6.10	5.15	4.64
**Vegetable preserves:**	**1.62**	**1.29**	**1.35**	**3.04**	**2.4**	**2.01**	**1.47**
Frozen vegetables and mushrooms	0.28	0.21	0.04	0.57	0.43	0.35	0.28
Sour cabbage	0.37	0.09	0.40	0.47	0.28	0.16	0.14
Other vegetable and mushroom preserves	0.97	0.99	0.91	2.00	1.70	1.51	1.05
**Potatoes and potato products:**	**0.84**	**5.19**	**0.22**	**16.23**	**5.54**	**8.53**	**3.63**
Potatoes	0.68	4.72	0.18	15.07	5.00	7.87	3.27
Potato products	0.16	0.47	0.04	1.16	0.54	0.67	0.36

The values in the columns do not add up because each time the conversion is done from the whole population according to the R environment rule. Differences are in hundredths of decimal places as a result of calculations from a database with many thousands of records. Data in bold are cumulative for the entire population or summed for individual groups, i.e., vegetables, vegetable products, and potatoes and potatoes products.

**Table 4:** Vegetables, potatoes and their products as sources of vitamins in the diet.

Specification	Thiamin	Riboflavin	Niacin	Vitamin B6	Folate	Vitamin C	Vitamin A	Vitamin D	Vitamin E
**Average daily supply**	**1.32 mg**	**1.59 mg**	**16.21 mg**	**1.84 mg**	**275.02 µg**	**91.40 mg**	**1194.55 µg**	**4.61 µg**	**13.45 mg**
**Supply from vegetables, potatoes and their products**	**0.23 mg**	**0.18 mg**	**2.64 mg**	**0.51 mg**	**85.20 µg**	**47.37 mg**	**365.17 µg**	**0.96 µg**	**1.61 mg**
**Share of vegetables, potatoes and their products in average daily supply (%):**	**17.11**	**11.26**	**16.32**	**27.76**	**30.98**	**51.81**	**30.57**	**20.80**	**11.96**
** Vegetables:**	**9.02**	**6.80**	**6.21**	**10.03**	**21.41**	**30.60**	**25.73**	**20.34**	**8.07**
Lettuce	0.18	0.28	0.07	0.17	1.35	0.74	0.93	0.00	0.19
Cabbage	0.68	0.76	0.41	1.05	2.84	7.03	1.12	0.00	1.35
Cauliflower	0.25	0.22	0.13	0.27	0.80	2.37	0.12	0.00	0.12
Tomatoes	1.42	0.77	1.84	2.36	3.97	7.35	2.91	0.00	3.04
Cucumber	0.24	0.28	0.17	0.20	0.59	0.96	0.31	0.00	0.12
Carrots	0.53	0.44	0.37	1.05	1.46	0.54	16.34	0.00	0.57
Beetroot	0.11	0.18	0.13	0.16	1.28	0.77	0.44	0.00	0.12
Onions	0.29	0.24	0.16	0.69	0.75	0.89	0.02	0.00	0.13
Other vegetables and mushrooms	5.32	3.63	2.94	4.07	8.36	9.95	3.54	20.34	2.42
** Vegetable products:**	**1.45**	**1.50**	**1.49**	**2.44**	**3.46**	**7.11**	**4.64**	**0.33**	**2.80**
Frozen vegetables and mushrooms	0.36	0.28	0.33	0.5	1.19	1.60	1.68	0.02	0.34
Sour cabbage	0.13	0.22	0.04	0.53	0.34	1.17	0.02	0.00	0.00
Other vegetable and mushroom products	0.96	0.99	1.13	1.40	1.93	4.33	2.95	0.31	2.46
** Potatoes and potato products:**	**6.64**	**2.96**	**8.62**	**15.29**	**6.12**	**14.10**	**0.19**	**0.13**	**1.10**
Potatoes	6.16	2.61	7.84	14.27	5.72	13.35	0.09	0.00	0.41
Potato products	0.48	0.36	0.78	1.02	0.39	0.75	0.10	0.13	0.68

The values in the columns do not add up because each time the conversion is done from the whole population according to the R environment rule. Differences are in hundredths of decimal places as a result of calculations from a database with many thousands of records. Data in bold are cumulative for the entire population or summed for individual groups, i.e., vegetables, vegetable products, and potatoes and potatoes products.

**Table 5:** Cluster analysis: impact of household characteristics on the energy and nutrient supply from vegetable, potatoes, and their products.

Factors	Cramer Correlations
Education level	**0.164**
Income (quintile group)	**0.163**
Village size	**0.150**
Socio-economic affiliation	**0.146**
Urbanisation degree	**0.131**
Land use	**0.127**
Study month	0.115
Financial status self-assessment	0.108
Household size	0.092
Nutritional self-assessment	0.086
Region	0.083
Family life phase	0.080
Gender	0.059
Age	0.054

The most influential factors are highlighted in bold.

**Table 6:** Cluster analysis: characteristics of clusters in terms of selected socio-economic characteristics.

**Specification**	**Sample Population**	**Cluster 1**	**Cluster 2**	**Cluster 3**
	100%	55.7%	27.1%	17.2%
Number of households	36,886	20,538	9992	6356
Number of persons in households	99,230	55,798	28,540	14,892
**Factors**	**% of the Sample Population**	**% of the Cluster Population**		
**Education Level**				
Lower secondary, primary	13.7%	12.4%	19.7%	8.4%
Basic vocational	31.3%	30.8%	39.2%	2.3%
Secondary and post-secondary	32.6%	33.6%	29.4%	34.8%
higher	22.4%	23.3%	11.7%	36.5%
**Income (Quintile Group)**				
First group	20.0%	18.6%	28.8%	10.6%
Second group	20.0%	19.9%	23.8%	14.4%
Third group	20.0%	20.4%	19.9%	18.8%
Fourth group	20.0%	20.6%	17.0%	22.8%
Fifth group	20.0%	20.5%	10.5%	33.3%
**Village Size**				
500,000 residents and more	12.9%	13.3%	6.3%	21.9%
200,000 to 499,000 residents	8.5%	9.1%	5.7%	11.0%
100,000 to 199,000 residents	7.9%	8.6%	6.2%	8.3%
20,000 to 99,000 residents	17.2%	18.0%	15.1%	18.1%
Less than 20,000 residents	11.2%	11.8%	11.0%	9.9%
Village	42.3%	39.3%	55.8%	30.9%
**Socio-Economic Affiliation**				
White-collar workers	24.5%	25.6%	27.9%	15.5%
Employees in manual labour positions	24.0%	25.7%	14.6%	33.3%
Farmers	4.6%	3.5%	8.2%	2.3%
Self-employed	6.8%	7.1%	4.7%	9.0%
Pensioners	29.9%	28.3%	32.3%	31.0%
Retired	6.3%	5.9%	7.6%	5.5%
Recipients of social benefits	2.6%	2.4%	3.7%	1.5%
Living from other unearned sources	1.5%	1.6%	1.1%	1.8%
**Urbanization Degree**				
Densely populated area	35.4%	37.1%	24.1%	47.7%
Medium populated area	22.9%	23.7%	21.7%	22.1%
Sparsely populated area	41.7%	39.1%	54.2%	30.2%
**Land Use**				
Yes	52.6%	49.3%	63.0%	47.3%
No	47.4%	50.7%	37.1%	52.7%

**Table 7:** Cluster analysis: percentage contribution of energy, macronutrients, minerals, and vitamins from vegetables, potatoes, and their products.

Specification	Sample Population	Cluster 1	Cluster 2	Cluster 3
Energy	7.33%	5.13%	10.92%	9.08%
Protein	7.93%	5.89%	10.21%	11.96%
Carbohydrates	12.58%	8.73%	19.54%	15.25%
Total fat	2.03%	1.96%	1.67%	2.83%
Fibre	31.80%	26.33%	39.16%	40.91%
Calcium	9.35%	7.69%	11.69%	14.49%
Phosphorus	12.20%	8.78%	17.16%	16.83%
Sodium	3.82%	3.05%	4.01%	5.32%
Potassium	32.45%	24.83%	45.93%	37.76%
Iron	19.00%	14.67%	23.67%	27.52%
Magnesium	20.15%	14.76%	28.21%	26.03%
Zinc	12.98%	9.81%	16.09%	20.14%
Thiamin	17.11%	12.66%	22.68%	24.71%
Riboflavin	11.26%	8.66%	14.04%	17.03%
Niacin	16.32%	11.65%	23.49%	20.47%
Vitamin B6	27.76%	20.49%	40.98%	32.69%
Folate	30.98%	25.65%	36.67%	43.83%
Vitamin C	51.81%	45.13%	65.22%	57.17%
Vitamin A	30.57%	25.92%	32.61%	43.09%
Vitamin D	20.80%	17.02%	17.93%	39.53%
Vitamin E	11.96%	10.84%	11.59%	18.70%

## Data Availability

Data are available at the Department of Food Market and Consumption research due to annual agreements signed between the Institute of Human Nutrition, Warsaw University of Life Sciences and Statistics Poland in Warsaw.
